# Oxidative Stress Correlates with Headache Symptoms in Fibromyalgia: Coenzyme Q_10_ Effect on Clinical Improvement

**DOI:** 10.1371/journal.pone.0035677

**Published:** 2012-04-19

**Authors:** Mario D. Cordero, Francisco Javier Cano-García, Elísabet Alcocer-Gómez, Manuel De Miguel, José Antonio Sánchez-Alcázar

**Affiliations:** 1 Centro Andaluz de Biología del Desarrollo (CABD), Universidad Pablo de Olavide-CSIC-Junta de Andalucía and Centro de Investigación Biomédica en Red de Enfermedades Raras (CIBERER), ISCIII, Sevilla, Spain; 2 Departmento Citología e Histología Normal y Patológica, Facultad de Medicina, Universidad de Sevilla, Sevilla, Spain; 3 Departmento de Personalidad, Evaluación y Tratamiento Psicológicos. Facultad de Psicología Universidad de Sevilla, Sevilla, Spain; California Pacific Medical Center Research Institute, United States of America

## Abstract

**Background:**

Fibromyalgia (FM) is a chronic pain syndrome with unknown etiology and a wide spectrum of symptoms such as allodynia, debilitating fatigue, joint stiffness and migraine. Recent studies have shown some evidences demonstrating that oxidative stress is associated to clinical symptoms in FM of fibromyalgia. We examined oxidative stress and bioenergetic status in blood mononuclear cells (BMCs) and its association to headache symptoms in FM patients. The effects of oral coenzyme Q_10_ (CoQ_10_) supplementation on biochemical markers and clinical improvement were also evaluated.

**Methods:**

We studied 20 FM patients and 15 healthy controls. Clinical parameters were evaluated using the Fibromyalgia Impact Questionnaire (FIQ), visual analogues scales (VAS), and the Headache Impact Test (HIT-6). Oxidative stress was determined by measuring CoQ_10_, catalase and lipid peroxidation (LPO) levels in BMCs. Bioenergetic status was assessed by measuring ATP levels in BMCs.

**Results:**

We found decreased CoQ_10_, catalase and ATP levels in BMCs from FM patients as compared to normal control (P<0.05 and P<0.001, respectively) We also found increased level of LPO in BMCs from FM patients as compared to normal control (P<0.001). Significant negative correlations between CoQ_10_ or catalase levels in BMCs and headache parameters were observed (r = −0.59, P<0.05; r = −0.68, P<0.05, respectively). Furthermore, LPO levels showed a significant positive correlation with HIT-6 (r = 0.33, P<0.05). Oral CoQ_10_ supplementation restored biochemical parameters and induced a significant improvement in clinical and headache symptoms (P<0.001).

**Discussion:**

The results of this study suggest a role for mitochondrial dysfunction and oxidative stress in the headache symptoms associated with FM. CoQ10 supplementation should be examined in a larger placebo controlled trial as a possible treatment in FM.

## Introduction

Fibromyalgia (FM) is a common chronic pain syndrome with an unknown etiology, which has been associated with a wide spectrum of symptoms such as allodynia, debilitating fatigue, joint stiffness and depression. FM is diagnosed according to the classification criteria established by the American College of Rheumatology (ACR) [1]. Despite being a common disorder that affects at least 5 million individuals in the United States [2], its pathogenic mechanism remains elusive. In addition to the described symptoms, a high prevalence of FM has been found among patients with transformed migraine and headaches [3], [4]. Common genetic basis, synergetically working with other factors (emotional, personality features, stressful events and medication overuse) should cause a chronic antinociceptive system alteration and therefore a progressive increase (hyperalgesia) and diffusion (panalgesia) of pain. It has been hypothesized that episodic migraine, chronic daily headaches and FM may actually be a continuum of the same disorder [5].

Recently, oxidative stress has been proposed as a relevant event in the pathogenesis of FM and headaches [6], [7]. Previously, our group has detected decreased coenzyme Q_10_ (CoQ_10_) levels and increased mitochondrial reactive oxygen species (ROS) production in blood mononuclear cells (BMCs) from FM patients [8]. Furthermore, oxidative stress showed a significant correlation with clinical symptoms in FM [6].

CoQ_10_ levels and mitochondrial dysfunction have also been implicated in the pathophysiology of migraine, and it has been reported that oral CoQ_10_ supplementation improved clinical symptoms [9].

The aim of this paper was first to establish a possible correlation between oxidative stress parameters and severity of headaches in FM, and secondly to study the effects of oral CoQ_10_ supplementation on the improvement in headache symptoms.

## Patients and Methods

### Ethics Statement

Written informed consent and the approval of the ethical committee of University Pablo de Olavide and Universitary Hospital Virgen Macarena from Seville were obtained, according to the principles of the Declaration of Helsinki.

### Patients

All samples were obtained after informed consent from patients and the approval of the local ethical committee was obtained according to the principles of the Declaration of Helsinki. The study consisted of 20 women diagnosed with FM and 15 healthy women. The inclusion criteria was fibromyalgia that had been diagnosed for the previous 2 to 3 years, based on the current ACR diagnostic criteria ^1^. Exclusion criteria were acute infectious diseases in the previous 3 weeks; past or present neurological, psychiatric, metabolic, autoimmune, allergy-related, dermal or chronic inflammatory disease; undesired habits (e.g., smoking, alcohol, etc.); oral diseases (e.g., periodontitis); medical conditions that required glucocorticoid treatment, use of analgesics, statin or antidepressant drugs; past or current substance abuse or dependence and pregnancy or current breastfeeding. Healthy controls had no signs or symptoms of FM and were free of any medication for at least 3 weeks before the study began. All patients and controls had taken no drugs or vitamin/nutritional supplement during the 3 week period prior to the collection of the blood samples. Before the study, the patients reported using paracetamol on demand. Clinical data was obtained from physical examination, and evaluated using the Fibromyalgia Impact Questionnaire (FIQ) including visual analogues scales about general and diffuse pain typical of FM (VAS), and Headache Impact Test (HIT-6).

### Blood mononuclear cells

Heparinized blood samples were collected after 12-hours fasting from patients and healthy age and sex-matched control subjects. BMCs were purified from heparinized blood by isopycnic centrifugation using Histopaque-1119 and Histopaque-1077 (Sigma Chemical Co., St. Louis, MO, USA).

### Measurement of CoQ10 levels

CoQ_10_ content in BMCs were analyzed by HPLC (Beckman Coulter, Brea, CA, USA; 166-126 HPLC) with ultraviolet detection (275 nm), according to the method described above [8].

### Lipid peroxidation

Lipid peroxidation in cells was determined by analyzing the accumulation of lipoperoxides using a commercial kit from Cayman Chemical (Ann Arbor, Michigan, USA). TBARS are expressed in terms of malondialdehyde (MDA) levels. In these assays, an MDA standard is used to construct a standard curve against which unknown samples can be plotted.

### Catalase determination

A spectrophotometric method described by Beer and Sizer (1952) [10] was used for measuring the breakdown of hydrogen peroxide by catalase. Briefly, activity was determined by using 35 µg of cell lysate, prepared in a lysis buffer composed of 0.9% NaCl, 20 mM Tris.ClH, pH = 7.6, 0.1% triton X-100, 1 mM phenylmethylsulfonylfluoride and 0.01% leupeptine with gentle shaking, in a kinetic spectrophotometric assay that measures a decrease in the absorbance of hydrogen peroxide.

### ATP levels

ATP levels were determined by a bioluminescence assay using an ATP determination kit from Invitrogen-Molecular Probes (Eugene, OR, USA) according to the instructions of the manufacturer.

### Oral CoQ_10_ supplementation

Ten volunteer patients were supplemented with CoQ_10_ (Pharma Nord, Vejle, Denmark) with soft gel capsules for 3 months (300 mg/day CoQ_10_ divided in three doses). After 3 months of treatment, heparinized blood samples were collected after 12-hours fasting and 24 hours after the last dose, and clinical symptoms were evaluated. The CoQ_10_ formulation consisted of soft gelatin capsules containing 100 mg of ubiquinone emulsified with diglyceryl monooleate, beeswax, soy lecithin and canola oil.

### Statistical Analysis

All results are expressed as mean ± SD unless stated otherwise. The unpaired Student's t test was used to evaluate the significance of differences between groups. Statistical analyses included Pearson's correlations between CoQ_10_, catalase, and MDA levels in compared with Hit-6 score. P values less than 0.05 were considered significant. Data were analysed using the SPSS/PC statistical software package (SPSS for Windows, 19, 2010, SPSS Inc. Chicago, IL, USA).

## Results

### Oxidative stress in FM

The mean age of patients was 46.6±5 years for the FM group and 44.9±4 years for the control group. The mean duration of symptoms in the FM group was 10.1±4.2 years. The mean tender point score in the FM group was 14.8±1.7 points. According to International Headache Society (IHS) criteria, the headache was tension-type headache. The mean of frequency of headache was 3±1 per week. The duration of headache episodes was 10±2 hours. The patients did not describe any symptoms such as nausea, photophobia, or aura.

The most prominent features of these FM patients were pain and stiffness. They were sedentary people and routine laboratory tests yielded normal results for glucose, urea, uric acid, total protein, creatinine, aspartate aminotransferase, alanine aminotransferase, cholesterol and triglycerides (data not shown). The number and subgroup distribution of BMCs (monocytes and limphocytes) in FM patients were in the normal range (data not shown).

**Figure 1:**
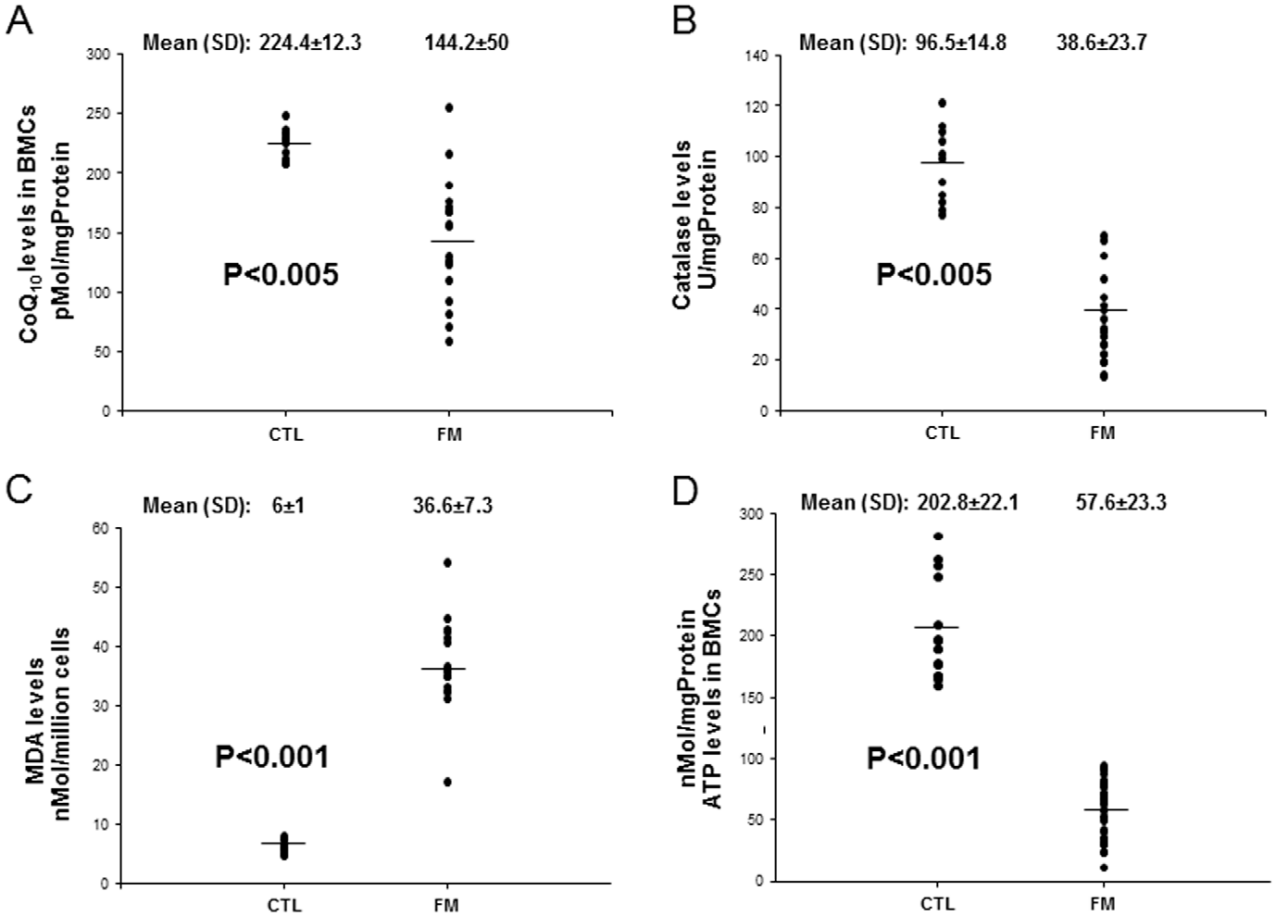
Coenzyme Q_10_ levels, catalase levels and lipid peroxidation (MDA levels) in blood mononuclear cells (BMCs) from fibromyalgia (FM) patients and healthy control individuals. (A) CoQ_10_ levels were measured by HPLC, as described in Materials and Methods. (B) Catalase was analyzed in BMCs as described in Materials and Methods. (C) LPO was measured as described in Material and Methods. (D) ATP levels were analyzed in BMCs as described in Materials and Methods. Data represent the mean ± SD of three separate experiments.

To evaluate the antioxidant system in FM patients, CoQ_10_ and catalase levels in BMCs were examined and compared to control subjects. Both, CoQ_10_ and catalase levels were significantly reduced in FM patients, 64.3% and 40% respectively (Figure 1A and 1B). We also determined LPO levels in BMCs from FM patients as a marker of oxidative stress-induced membrane damage by ROS. FM patients showed higher LPO levels in BMCs, 610% with respect to control subjects (Figure 1C).

To determine whether the observed CoQ_10_ deficiency had an effect on cellular bioenergetics, we measured intracellular ATP levels in BMCs from control and FM patients. ATP levels were reduced to 70% of the control value in BMCs from FM patients (Figure 1D).

### Headache correlates with oxidative stress in FM patients

**Figure 2:**
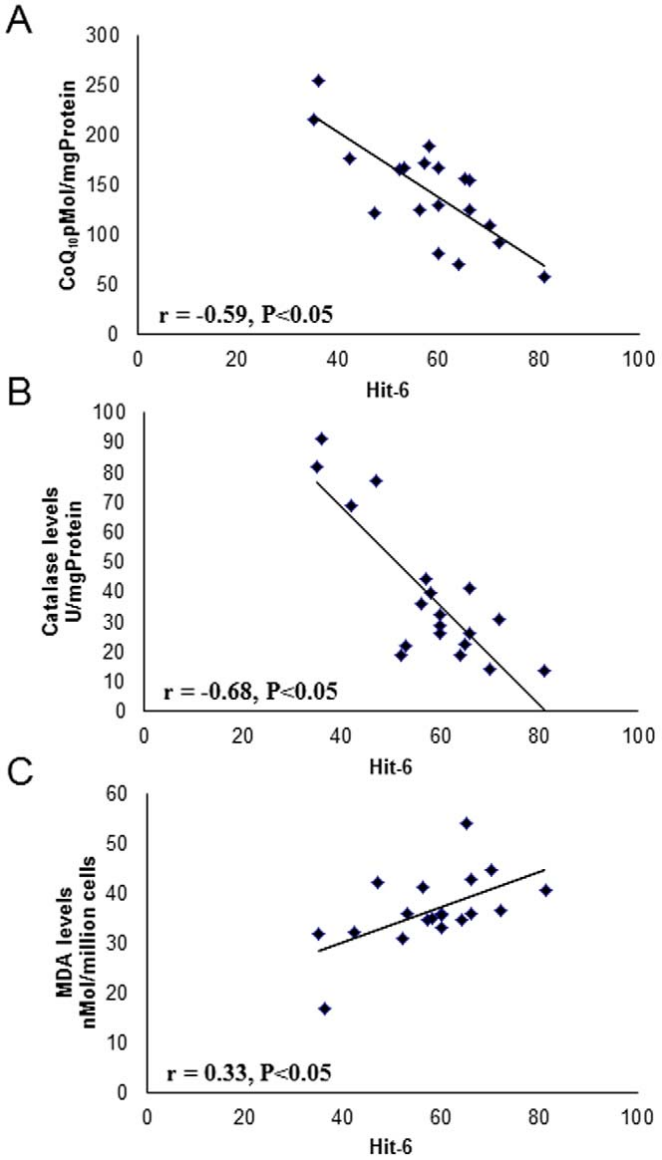
Correlation of CoQ_10_, catalase or lipid peroxidation levels in BMCs from FM patients and HIT-6 levels.

All FM patients showed high HIT-6 scores compared with control subjects (Table 1). To examine whether headache symptoms were associated to increased oxidative stress, Pearson's correlation coefficients (r) were performed between CoQ_10_, catalase or LPO levels and HIT-6 scores. Figure 2 shows a significant negative correlation between CoQ_10_ and catalase levels and HIT-6 (r = −0.59, P<0.05; r = −0.68, P<0.05, respectively). Furthermore, LPO levels showed a significant positive correlation with HIT-6 (r = 0.33, P<0.05). No correlation of ATP levels and HIT-6 scores was found (data not shown).

**Table 1:** Characteristic finding of FM patients and control groups.

	Patients (n = 20)		Control (n = 15)	
Age (yr)	46.6	±5	44.9	±4
Tender points	14.8	±1.7	---	
Duration of disease (years)	10.1	±4.2	---	
FIQ Total score, range 0–100	60.2	±5.6*	7.1	±1.4
VAS, range 0–10	6.9	±1.3*	0.8	±0.3
HIT-6, range 36–78	61.8	±1.3*	36.1	±1.1

VAS: Visual Analogue Scale; FIQ: Fibromyalgia Impact Questionnaire; HIT-6: Headache Impact Test. Values are means ±SD,

* P<0.001.

### Influence of oral Coenzyme Q_10_ supplementation

Table 2 shows levels of biochemical markers in control subjects and before and after treatment with CoQ_10_. In FM patients treated with CoQ_10_, all levels of oxidative stress markers were similar to those measured in control subjects. CoQ_10_ supplementation significantly increased levels of CoQ_10_, ATP and catalase but all levels were lower than those seen in controls. In contrast, MDA in BMCs declined and fell to levels similar to those seen in controls. These biochemical marker changes were associated with improvement of clinical measured by tender points, FIQ VAS and HIT-6 scores.

**Table 2:** Clinical symptoms and biochemical markers after CoQ_10_ treatment.

	Pre-treatment (n = 10)	Post-treatment (n = 10)	Control levels (n = 15)
Tender points	13.9±1.2	9±0.5*	---
FIQ total score, range 0–100	59.2±4.2†	30.1±2*	7.1±1.4
VAS, range 0–10	7.1±1.2†	3.5±0.8*	0.8±0.3
HIT-6, range 36–78	60.8±1.4†	36.9±1.7*	36.1±1.1
CoQ_10_ (pmolQ/mg protein)	135.6±6.3†	221.6±11.3*	224.4±12.3
ATP (nmol/mg protein)	61.3±4.9†	191.1±6.7*	202.8±22.1
Catalase (U/mg protein)	35.6±10.1†	85.2±15.3*	96.5±14.8
MDA in BMCs (nmol/million cells)	30.3±5.9†	5.1±1.6*	6±1

VAS: Visual Analogue Scale; FIQ: Fibromyalgia Impact Questionnaire; HIT-6: Headache Impact Test; MDA: Malondialdehyde; BMCs: Blood mononuclear cells. Values are means ±SD,

* P<0.001 between pre and post treatment;

† P<0.001 between pretreatment and control.

We observed a significant increase of CoQ_10_ levels after treatment (135.6±6.3 pretreatment and 221.6±11.3 postreatment, P<0.001) (respect to control, 224.4±12.3;P<0.001), ATP levels (61.3±4.9 pretreatment and 191.1±6.7 postreatment, P<0.001) (respect to control, 202.8±22.1;P<0.001) and catalase levels (35.6±10.1 pretreatment and 85.2±15.3 postreatment, P<0.001) (respect to control, 96.5±14.8; P<0.001) in BMCs, a reduction of LPO levels (30.3±5.9 pretreatment and 5.1±1.6 postreatment, P<0.001) (respect to control, 6±1; P<0.001) and a marked improvement of clinical symptoms (FIQ: P<0.01; VAS: P<0.01; HIT-6: P<0.05) (Table 2). No biochemical alterations were detected after CoQ_10_ treatment: glucose 82,3±10,16 mg/dL (normal range: 76–110), urea 29,7±5,31 mg/dL (normal range: 10–45), uric acid 5,1±1,41 mg/dL (normal range: 2.5–7.5), total protein 7,2±1,01 g/dL (normal range: 6.6–8.7), creatinine 0,9±0,13 mg/dL (normal range: 0.5–1.1), aspartate aminotransferase 24,3±5,17 mU/mL (normal range: 10–40), alanine aminotransferase 21,2±8,11 mU/mL (normal rage: 10–40), total cholesterol 205±9,01 mg/dL (normal range: <220), and triglycerides 168±31,13 mg/dL (normal range: 150–200).

## Discussion

In the present study we have confirmed a significant increase of oxidative stress in FM patients, showing a marked decrease of CoQ_10_, ATP and catalase levels and a significant increase of LPO levels in BMCs compared to control subjects.

Coenzyme Q_10_ (CoQ_10_) is present in every membrane of all cells in the body. CoQ_10_ transfers electrons from complexes I and II to complex III in the mitochondrial respiratory chain and fulfills a critical role in mitochondrial ATP production, playing a crucial role in cellular metabolism; regulating mitochondrial uncoupling proteins, the mitochondrial permeability transition pore, β-oxidation of fatty acids, nucleotide metabolism and production of reactive oxygen species (ROS) [11], [12]. It has been widely demonstrated that CoQ_10_ is essential for respiratory chain efficacy, and as antioxidant [13], [14], [15]. CoQ_10_ deficiency has been associated with several diseases with the typical symptoms found in FM patients [16], [17]. Interestingly, CoQ_10_ deficiency has been detected in depression and chronic fatigue [18], [19], two typical symptoms found in FM patients. Furthermore, both symptoms were markedly improved after CoQ_10_ supplementation. Our results are in agreement with those of previous reports which have shown that CoQ deficiency is associated with decreased ATP levels and increased oxidative stress [20], [21]. Oxidative stress and, in particular, LPO levels have been implicated in the severity of the clinical symptoms in FM and it has been suggested that antioxidant therapy could be beneficial in FM [6]. On the other hand, the oxidant-antioxidant balance disorders underlie a number of acute and chronic diseases of the central nervous system (CNS) [22]. Clinical conditions affecting the nervous system range from mild cognitive perturbations such as headache or migraine, to life-threatening acute courses such as meningitis, and to chronic neurodegenerative diseases such as multiple sclerosis. One common feature in clinical dysfunctions within the nervous system is redox regulation, with an imbalance in oxidative stress versus antioxidants being characteristic of pathological conditions. It is believed that oxidative stress and LPO play a role in the pathogenesis of migraine by regulating cerebral blood flow and energy metabolism and may constitute a trigger threshold for migraine attacks [23], [24]. Moreover, emerging data suggest that LPO may underlie the neuronal alterations and neurotoxicity observed in numerous neuropathological conditions. Direct application of LPO, either *in vivo* or *in vitro*, has been shown to be cytotoxic and to mimic neuronal alterations observed in neuropathological conditions. In addition, prevention of LPO has been demonstrated to be neuroprotective in a variety of neuropathological paradigms [25]. It is known that LPO, as a consequence of oxidative stress, indirectly reflects intracellular ROS generation, and ROS are known to be implicated in the etiology of pain, one of the most prominent symptoms in FM, by inducing peripheral and central hyperalgesia [26].

Our results show an important significant correlation between LPO levels and headache symptoms in FM patients. Antioxidants (CoQ_10_ and catalase) levels also showed a significant negative correlation with headache symptoms. CoQ_10_ has two important functions in cells: first, CoQ_10_ is a mitochondrial cofactor with the potential to boost mitochondrial function, and second, CoQ_10_ is a powerful free radical scavenger that can mitigate lipid peroxidation and DNA damage caused by oxidative stress [27]. In our study, we have observed a marked improvement in headache symptoms and a significant recovery in oxidative stress markers after CoQ_10_ supplementation, suggesting that CoQ_10_ may be involved in the pathophysiology of headache symptoms in FM.

Mitochondria have long been postulated to be involved in the etiology of migraines, although a direct link has not been identified [28]. In addition, the inflammatory component of migraines may produce oxygen free radicals, consuming CoQ_10_ and inducing CoQ_10_ deficiency [29].

Clinical studies have generated evidence that FM is associated with immune dysregulation of circulatory levels of pro-inflammatory cytokines, affecting neural function of pain-related neurotransmitters [30]. Cytokines, depending on their concentration, induce symptoms, such as fatigue, fever, sleep, pain, and myalgia [31], all of which develop in FM patients. Alterations in pro-inflammatory cytokine levels have been observed in the serum and skin biopsies of FM patients [32], [33]. Interestingly, several studies have also suggested that inflammatory processes are involved in the physiopathology of migraine [34], [35]. The inflammatory and pain component of FM and migraine is behind the rationale of using nonsteroidal anti-inflammatory drugs (NSAIDs) for the treatment of both symptoms. However, while the use of NSAIDs for fibromyalgia is a fairly common practice, little objective evidence is available upon which to assess the efficacy of these agents [36]. There are multiple mechanisms of action upon which the NSAIDs act. There have been conflicting reports as to whether NSAIDs such as acetylsalicylic acid are effective in protecting neurons against neurotoxicity. Acetaminophen has been shown to rescue neuronal cells from mitochondrial redox impairment, lipoperoxidative products and MDA generation [37]. Furthermore, acetaminophen also reduced the cytoplasmic accumulation of peroxides. Acetylsalicylic acid and acetaminophen inhibit lipid peroxidation and cell damage, in vivo, in the rat hippocampus [38]. The results obtained when NSAIDs are combined with benzodiazepines have also been inconsistent [39]. However, NSAIDs can be helpful in reducing pain flares induced by excessive physical activity, tendinitis or bursitis, although they should only be used on an as needed basis in order to avoid side effects [40].

In addition of pro-inflammatory cytokines, CoQ_10_ deficiency is also involved in inflammation. A significant negative correlation has been observed between CoQ_10_ and pro-inflammatory markers in septic shock patients [41], and expression profiling revealed that CoQ_10_ influences the expression of inflammatory genes suggested that CoQ_10_ exerts anti-inflammatory properties. In another study, administration of CoQ_10_ significantly attenuated the increase of oxidative and nitrative stress markers and inflammatory markers in an animal model of metabolic syndrome [42].

In summary, headache symptoms in FM could be a consequence of oxidative stress and both may share common pathophysiologic basis. Furthermore, CoQ_10_ treatment showed a remarkable improvement in clinical symptoms and headache in FM. Detection of CoQ_10_ deficiency and subsequent CoQ_10_ supplementation may result in clinical improvement in FM. Further analysis involving doubled-blind placebo-controlled clinical trials will be required to confirm this observation.
